# Clinicopathological significance of expression of p-c-Jun, TCF4 and beta-Catenin in colorectal tumors

**DOI:** 10.1186/1471-2407-8-328

**Published:** 2008-11-08

**Authors:** Kayoko Takeda, Ichiro Kinoshita, Yasushi Shimizu, Yusuke Ohba, Tomoo Itoh, Yoshihiro Matsuno, Toshiaki Shichinohe, Hirotoshi Dosaka-Akita

**Affiliations:** 1 Department of Medical Oncology, Hokkaido University Graduate School of Medicine, North 15, West 7, Kita-ku, Sapporo 060-8638, Japan; 2 Laboratory of Pathophysiology and Signal Transduction, Hokkaido University Graduate School of Medicine, North 15, West 7, Kita-ku, Sapporo 060-8638, Japan; 3 Department of Surgical Pathology, Hokkaido University Hospital, North 14, West 5, Kita-ku, Sapporo 060-8648, Japan; 4 Department of Surgical Oncology, Hokkaido University Graduate School of Medicine, North 15, West 7, Kita-ku, Sapporo 060-8638, Japan

## Abstract

**Background:**

A recent study has shown that phosphorylated c-Jun (p-c-Jun) interacts with TCF4 to form a complex that cooperatively enhances their transcriptional activity in the presence of β-Catenin, and that their interaction is critical for mouse intestinal tumorigenesis. To determine the significance of these three proteins in human colorectal tumors, we analyzed their nuclear expression by immunohistochemistry.

**Methods:**

we analyzed their nuclear expression by immunohistochemistry using paraffin-embedded specimens of 68 resected colorectal tumors, which consisted of 19 adenomas, 14 high-grade intraepithelial neoplasia (HGINs) and 35 adenocarcinomas. We also analyzed the expression of MMP7, which has functional AP-1 and TCF binding sites in its promoter.

**Results:**

Expression of p-c-Jun, TCF4 and β-Catenin were significantly higher in adenomas than in the adjacent normal epithelia. Expression of p-c-Jun and β-Catenin in HGINs and adenocarcinomas were also significantly higher than in the adjacent normal epithelia. p-c-Jun expression, but not TCF4 and β-Catenin, was higher in adenomas and HGINs than in adenocarcinomas, in which p-c-Jun expression was negatively correlated with pT stage progression. Furthermore, significant correlations of expression were observed between p-c-Jun and TCF4 (r = 0.25, p = 0.04), TCF4 and β-Catenin (r = 0.30, p = 0.01), p-c-Jun and MMP7 (r = 0.26, p = 0.03), and TCF4 and MMP7 (r = 0.39, p = 0.0008), respectively.

**Conclusion:**

These results suggest that nuclear expression of p-c-Jun, TCF4 and β-Catenin have important roles in human colorectal tumor development and that p-c-Jun may play a pivotal role in the earlier stages of tumor development.

## Background

Colorectal tumorigenesis is a multistep process involving genetic alterations of oncogenes and tumor suppressor genes, which lead to deregulation of a number of critical molecular pathways [1,2]. One of the important genetic abnormalities is the mutation of the adenomatous polyposis coli (*Apc*) tumor suppressor gene, which results in deregulation of the APC/β-Catenin/T-cell factor 4 (TCF-4) signaling pathway [1-3]. β-Catenin is a 92 kDa protein that binds to the cytoplasmic tail of E-cadherin and plays an important role in the Wnt signal transduction [4,5]. When mutations in the *Apc *gene or the β-Catenin gene itself occur, or when the Wnt pathway is up-regulated, β-Catenin accumulates in the cytoplasm and migrates to the nucleus [6]. The aberrantly accumulated β-Catenin in the nucleus activates transcription by forming a complex with the TCF/LEF HMG box transcription factors family, including the TCF4, with the net result of the activation of the target genes that include *c-myc, cyclin D1 *and *c-jun *[2,6,7].

The proto-oncoprotein c-Jun is a major component of dimeric AP-1 transcription factor [8]. Deregulated expression of c-Jun transforms rodent fibroblasts [9] depending on the induction of multiple c-Jun target genes [10-12], while the cell transformation by various oncogenes requires an increase of the c-Jun expression [13]. An important mechanism in the stimulation of the AP-1 function is amino-terminal phosphorylation of c-Jun by the c-Jun N-terminal kinases (JNKs) [14,15]. Phosphorylated c-Jun (p-c-Jun) is involved in stress-induced apoptosis, cellular proliferation and tumorigenesis [16]. A recent study has shown that p-c-Jun interacts with TCF4 to form a complex that cooperatively enhances the transcriptional activity in the presence of β-Catenin and that the interaction dependent on the phosphorylation of c-Jun is critical for mouse intestinal tumorigenesis to occur [17]. In addition, another recent study also shows physical and functional cooperation between AP-1 proteins including c-Jun and β-Catenin for the regulation of TCF-dependent genes [18]. However, expressions of p-c-Jun in combination with TCF4 and β-Catenin have not been explored with regard to human colorectal tumors.

To determine the significance of p-c-Jun, TCF4 and β-Catenin in human colorectal tumors, we analyzed the nuclear expression of these proteins in resected colorectal tumors by immunohistochemistry. We also analyzed the expression of MMP7, which has functional AP-1 and TCF binding sites in its promoter [19].

## Methods

### Tumor specimens

Primary tumor specimens from 68 colorectal tumors were consecutively obtained by surgery or endoscopically from the Hokkaido University Medical Hospital between 2000 and 2003. Informed consent from patients were obtained for the use of resected tumor specimens. The patients with colorectal tumors consisted of 35 men and 33 women, with an average age at diagnosis of 63.8 years. The tumor classifications were determined according to the guidelines of the International Agency for Research on Cancer (IARC) and World Health Organization (WHO) [20]. Tumor specimens were histopathologically diagnosed as adenomas (n = 19), high-grade intraepithelial neoplasia (HGINs) (n = 14) and adenocarcinomas (n = 35). The pTNM classifications were determined according to the guidelines of the International Union Against Cancer (UICC) [21]. Adenocarcinoma represented 9 pT_1_, 2 pT_2_, 19 pT_3 _and 5 pT_4 _stage tumors, and 20 pN_0_, 7 pN_1_, 6 pN_2 _and 2 pN_3 _stage tumors.

### Antibodies

The primary antibodies used for immunohistochemistry and immunofluorescence analyses included a rabbit polyclonal antibody to p-c-Jun (Phospho-c-Jun (Ser63) II; Cell Signaling, Cumming Center Beverly, MA) which recognizes c-Jun phosphorylated at Ser 63 [22,23], a mouse anti-β-Catenin monoclonal antibody (IgG1, Clone 14; Transduction Laboratories, San Diego, CA) [24-28], a mouse anti-Tcf-4 monoclonal antibody (IgG2a, 6H5-3; Sigma, St. Louis, Missouri) [29] and a mouse anti-human MMP7 monoclonal antibody (IgG1, Clone 141-7B2; Daiichi Fine Chemical, Toyama, Japan) [30,31].

### Immunohistochemistry analysis

Expression of p-c-Jun, TCF4, β-Catenin, and MMP7 was analyzed by immunohistochemistry. The avidin-biotin-peroxidase complex (ABC) method was used on 4-μm sections of formalin-fixed, paraffin-embedded tissues after deparaffinization. Briefly, deparaffinized tissue sections were microwaved in 0.01 M sodium citrate (pH 6.0) to retrieve the antigenicity for 25 min for p-c-Jun or 20 min for TCF4, β-Catenin and MMP7. The slides were allowed to cool for an additional 20 min in citrate buffer. The sections were then incubated with 3% (w/v) H_2_O_2 _in methanol to inhibit endogenous peroxidase activity, followed by incubation with normal goat serum (Vectastain Elite ABC kit; Vector Laboratories, Burlingame, CA) for p-c-Jun or with normal horse serum (Vectastain Elite ABC kit) for β-Catenin, TCF4 and MMP7 for 30 min at room temperature to block the nonspecific antibody binding sites. The sections were consecutively reacted with a rabbit polyclonal antibody against p-c-Jun (1:50 dilution) or with a mouse monoclonal antibody against TCF4 (1:200 dilution), β-Catenin (1:3200 dilution), or MMP7 (1:400 dilution) at 4°C overnight. After washing, biotinylated goat anti-rabbit IgG antibody (Vectastain Elite ABC kit) for p-c-Jun or biotinylated horse anti-mouse IgG antibody (Vectastain Elite ABC kit) for β-Catenin, TCF4 and MMP7 was applied for 30 min. After washing, avidin-biotin-peroxidase complex (Vectastain Elite ABC kit) was applied for 30 min followed by peroxidase detection with a mixture of 3,3'-diaminobenzidine (DAB; Vector Laboratories). To determine specificity of immunostaining, serial sections were similarly processed except that primary antibodies were omitted. Sections were counterstained with methyl green.

### Immunohistochemical evaluation

Two investigators (K.T. and I.K.) separately and independently evaluated the immunohistochemical staining without knowledge of the clinical data. The results were evaluated as immunohistochemistry (IHC) score, where the IHC score = (percentage of positive cells (percentage score)) × (staining intensity [which was scored as 0 to 3]) [26,27,32]. Significant differences in interpretation were resolved in conference. Such differences in interpretation only occurred in a small subset of specimens; discordance more than 30% in percentage score is 24% in total specimens of three types of tumors and their adjacent normal epithelia, and discordance more than one staining intensity score is 3%. Interobserver agreement on four staining intensity scores was also evaluated using the linearly weighted kappa statistics [33]. The kappa value was 0.46 in all cases (data not shown) and interpreted as moderate agreement [33]. In cases without significant discordance, scores of two observer were averaged.

### Statistical analyses

Wilcoxon matched pairs signed ranks test was used to determine the differences between the tumors and the adjacent normal epithelia. The Mann-Whitney U test and Kruskal-Wallis test were used to determine the difference between two groups and among more than two groups, respectively. *p *< 0.05 was considered statistically significant. Spearman's rank correlation test was used to determine the correlation with staining intensity and percentage score.

### Immunofluorescence analysis

For the immunofluorescent staining of p-c-Jun, TCF4 and β-Catenin, the sections were subjected to antigen retrieval by heating in 0.01 M sodium citrate (pH 6.0) in a microwave oven for 25 min. The slides were allowed to cool for an additional 20 min in citrate buffer. The sections were permeabilized in PBS containing 0.2% Triton X-100 (PBT) for 20 min at room temperature. Nonspecific binding sites were blocked with normal goat serum for 30 min at room temperature. The sections were incubated at 4°C overnight with a combination of the primary rabbit polyclonal antibody against p-c-Jun (1:2 dilution) and one of the mouse monoclonal antibodies against TCF4 (1:4 dilution) and β-Catenin (1:60 dilution). After washing, the sections were incubated for 90 min at room temperature with the mixture of Alexa Fluor 488-conjugated goat anti-rabbit IgG antibody and Alexa Fluor 568-conjugated goat anti-mouse IgG antibody which were highly cross-absorbed (Invitrogen, Eugene, OR). The sections were then evaluated and photographed under a fluorescence microscope. To determine specificity of the immunofluorescent staining, serial sections were similarly processed except that primary antibodies were omitted.

## Results

### Nuclear expression of p-c-Jun, TCF4, and β-Catenin in colorectal adenomas, HGINs and adenocarcinomas compared with adjacent normal colon epithelia

**Figure 1:**
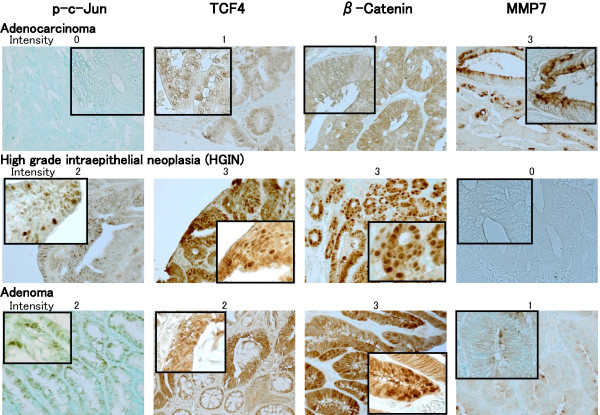
**Representative immunohistochemical staining patterns for p-c-Jun, TCF4, β-Catenin, and MMP7.** High-power view of representation area in each tumor is shown in a box. The staining intensity in the nucleus of tumor cells for p-c-Jun, TCF4 and β-Catenin and in the cytoplasm of the tumor cells for MMP7 was categorized as 0, 1, 2, or 3 (none, weak, moderate, or strong).

In the tumor cells of adenomas, HGINs and adenocarcinomas, staining for p-c-Jun was mainly detected in the nucleus, while staining was observed in the nucleus and cytoplasm for TCF4, and in the nucleus, cytoplasm and cellular membrane for β-Catenin. Staining for MMP7 was observed in the cytoplasm of the tumor cells. Representative immunostaining patterns for these proteins are shown in Fig. 1. Intensity of the nuclear stainings for p-c-Jun, TCF4 and β-Catenin, and that of the cytoplasmic staining for MMP7 were scored as 0, 1, 2, or 3 (none, weak, moderate, or strong). Significant immunostaining was not detected in serial sections when the primary antibodies were omitted (data not shown).

p-c-Jun, TCF4 and β-Catenin in the adenomas, and p-c-Jun and β-Catenin in the HGINs and adenocarcinomas showed significantly higher scores than in their adjacent normal colorectal epithelia, respectively (p < 0.05 by Wilcoxon matched pairs signed ranks test) (Additional file 1-Supplemental Table S1). TCF4 in HGINs also tended to have higher scores than in the adjacent epithelia.

Although we used IHC scores (see Methods) to evaluate results, high correlations were observed for each proteins between staining intensity and percentage score in adjacent normal epithelia and tumors of all three types (Additional file 2-Supplemental Table S2), which prompted us to see if the results would be similar when percentage score were used for evaluation. Using the percentage score, we observed similar results, and the difference of TCF4 between HGINs and adjacent normal epithelia also reached significance. (p < 0.05 by Wilcoxon matched pairs signed ranks test) (Additional file 3-Supplemental Table S3).

### Nuclear expression of p-c-Jun, TCF4, and β-Catenin among adenomas, HGINs and adenocarcinomas

**Figure 2:**
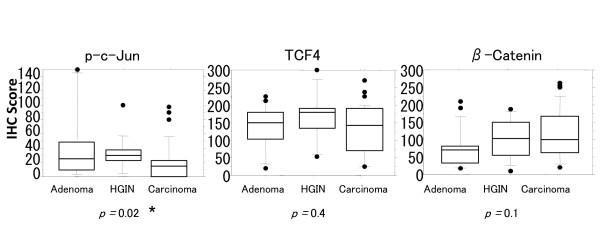
**Nuclear expression of p-c-Jun, TCF4 and β-Catenin in adenomas (n = 19), HGINs (n = 14) and adenocarcinomas (n = 35)**. Only p-c-Jun expression was significantly different. Adenomas and HGINs showed higher immunohistochemistry (IHC) scores than adenocarcinomas. Horizontal lines, median; boxes, 25% to 75% range; brackets, 10% to 90% range; circles, points outside the 10% to 90% range. *p < 0.05 by Kruskal-Wallis test.

Nuclear expression of p-c-Jun, TCF4 and β-Catenin was compared among the adenomas, HGINs and adenocarcinomas (Fig. 2). Only p-c-Jun expression was significantly different (p = 0.02 by Kruskal-Wallis test); adenomas and HGINs had higher scores than adenocarcinomas. There were no differences in the expression of TCF4 and β-Catenin among them. We observed the similar results when we used the percentage scores (p = 0.01 by Kruskal-Wallis test) (Additional file 4-Supplemental Figure S1).

### Relationship between nuclear expression of p-c-Jun, TCF4 and β-Catenin, and pT and pN stages in adenocarcinomas

**Figure 3:**
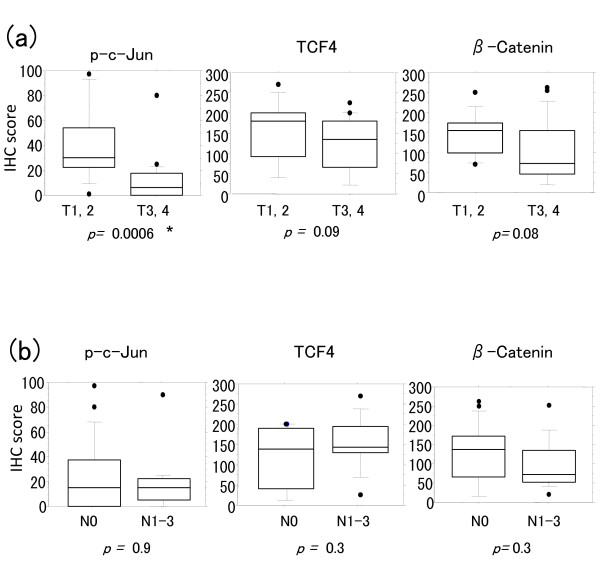
**Relationship between nuclear expression of p-c-Jun, TCF4 and β-Catenin and the pT and pN stages in adenocarcinomas (n = 35)**. a) p-c-Jun expression was significantly negatively correlated with the pT stage progression (T1-2, n = 11; T3-4, n = 24). b) No significant correlation was observed between p-c-Jun, TCF4 and β-Catenin expression, and the pN stage (N0, n = 20; N1-3, n = 15). Horizontal lines, median; boxes, 25% to 75% range; brackets, 10% to 90% range; circles, points outside the 10% to 90% range. *p < 0.05 by Mann-Whitney U test.

We analyzed the nuclear expression of p-c-Jun, TCF4 and β-Catenin in relation to the pT and pN stages in adenocarcinomas. We observed significantly lower p-c-Jun expression in tumors having the higher pT stage (p = 0.0006 by Mann-Whitney U test) (Fig. 3). Expression of TCF4 and β-Catenin was not significantly correlated with the pT stage. There was no significant correlation of expression of p-c-Jun, TCF4, and β-Catenin with the pN stage (Fig. 3). We observed the similar results including significantly lower p-c-Jun expression in tumors having the higher pT stage when we used percentage scores (p = 0.0009 by Mann-Whitney U test) (Additional file 5-Supplemental Figure S2).

### Correlation between the nuclear expression of p-c-Jun, TCF4 and β-Catenin, and cytoplasmic MMP7 expression

To investigate the relationships of p-c-Jun, TCF4, and β-Catenin with MMP7, whose promoter contains both functional AP-1 and TCF binding sites [19], we analyzed the correlation of the nuclear expression of p-c-Jun, TCF4 and β-Catenin with cytoplasmic MMP7 expression in colorectal tumors including adenomas, HGINs and adenocarcinomas. Significant correlations of exprssion were observed between p-c-Jun and TCF4 (r = 0.25, p = 0.04), and TCF4 and β-Catenin (r = 0.30, p = 0.01), p-c-Jun and MMP7 (r = 0.26, p = 0.03), and TCF4 and MMP7 (r = 0.39, p = 0.0008), respectively (Table 1).

**Table 1:** Correlation among expression of p-c-Jun, TCF, β-Catenin and MMP7 in all colorectal tumors (n = 68).

**Factors**	**R**	***p***
p-c-Jun/TCF4	0.25	0.04*
p-c-Jun/β-Catenin	0.13	0.3
p-c-Jun/MMP7	0.26	0.03*
TCF4/β-Catenin	0.30	0.01*
TCF4/MMP7	0.39	0.0008*
β-Catenin/MMP7	0.05	0.6

*p < 0.05 by Pearson's correlation test

### Immunofluorescence analysis of p-c-Jun, TCF4 and β-Catenin

**Figure 4:**
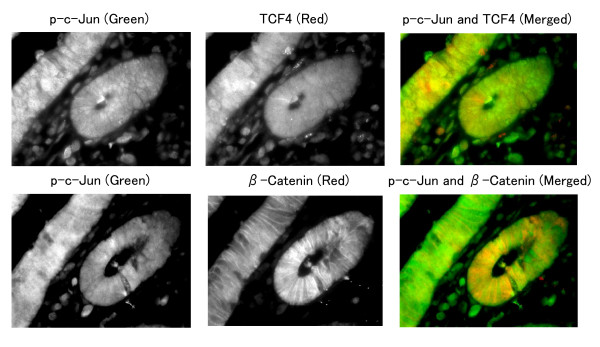
**Immunofluorescence analysis of p-c-Jun, TCF4 and β-Catenin in a representative specimen of adenocarcinoma**. Upper panels: p-c-Jun (Alexa 488; green) and TCF4 (Alexa 568; red) were colocalized predominantly in the nuclei in the majority of the tumor cells. Lower panels: β-Catenin (Alexa 568; red) was colocalized in some parts of the nuclei with p-c-Jun (Alexa 488; green), although β-Catenin was predominantly located in the cellular membrane of the tumor cells.

To determine whether p-c-Jun, TCF4 and β-Catenin are colocalized in the nuclei, several specimens of adenocarcinoma that expressed each protein were examined by immunofluorescence. A representative specimen demonstrated that p-c-Jun and TCF4 were colocalized predominantly in the nuclei in a majority of the tumor cells. Although β-Catenin was predominantly located in the cellular membrane of the tumor cells, it was also colocalized with p-c-Jun in some parts of the nuclei (Fig. 4). Significant immunofluorescent staining was not detected in serial sections when the primary antibodies were omitted (data not shown).

## Discussion

A recent study has shown that p-c-Jun interacts with TCF4 to form a complex that cooperatively enhances the transcriptional activity in the presence of β-Catenin, and that this interaction is critical for mouse intestinal tumorigenesis [17]. However, only a few studies have examined c-Jun, TCF4 and β-Catenin expression in human colorectal tumors [17,26-29]. In addition, there have been no studies that have analyzed their expression within the same specimens of human colorectal tumors. In the present study, we demonstrated that p-c-Jun, TCF4 and β-Catenin are frequently overexpressed in the nucleus of resected human colorectal tumors when compared to the adjacent normal epithelia by immunohistochemistry, suggesting that these three proteins may play important roles in human colorectal tumor development. The high expression of p-c-Jun in adenomas and HGINs, as compared with adenocarcinomas, and its negative correlation with pT stage progression in adenocarcinomas suggest that p-c-Jun may play a pivotal role in the earlier stages of tumor development. Furthermore, the multiple correlations among the expressions of these three proteins and MMP7, which is one of their downstream gene products, suggest that the interactions of p-c-Jun, TCF4 and β-Catenin that have been demonstrated in vitro [19] are also important in human colorectal tumor development.

Expression of c-Jun has been shown to be elevated in human colorectal adenomas and carcinomas when compared to adjacent normal epithelia [34]. c-Jun's transcriptional activity is enhanced through the N-terminal phosphorylation of c-Jun at the serin residues 63 and 73 by JNKs [16]. The present findings that the active phosphorylated form of c-Jun is also elevated in colorectal adenoma and carcinoma further support a potential role for c-Jun in human colorectal tumors.

While the published studies on c-Jun have not explored the correlations with pathological stages in adenocarcinoma [34], we have shown the higher p-c-Jun expression in adenomas and HGINs than in adenocarcinomas, and its negative correlation with pT stage progression in invasive cacinomas. The correlation of p-c-Jun expression with the earlier stages of human tumorigenesis is consistent with the recent in vivo studies for intestinal and liver tumors [17,35,36]. In the *Apc*^*Min *^mouse model of intestinal cancer, genetic abrogation of the c-Jun N-terminal phosphorylation or gut-specific conditional c-jun inactivation reduces tumor number and size, and prolongs the lifespan [17]. In the mouse model of the chemically induced hepatocellular carcinomas, c-jun is required for the early stages of tumor development, and the number and size of hepatic tumors is dramatically reduced by c-jun inactivation after the tumors have been initiated [35,36]. Furthermore, in human lung tumorigenesis, c-Jun is frequently overexpressed in an atypical area that includes dysplasia, and less frequently in lung cancer [37]. Taken together, these results suggest that c-Jun, especially in its phosphorylated state, may play a pivotal role in the early stages of the tumor development, including that of human colorectal tumors.

The similarly high levels of expression of TCF4 in the nucleus of the colorectal normal epithelia and adenocarcinomas are consistent with the results of previous studies by immunohistochemistry and in situ hybridization [29,38]. Interestingly, we found that the nuclear expression of TCF4 is significantly higher in adenomas than in the adjacent normal epithelia. Korinek et. al. [38] demonstrates that *Apc*^-/- ^colon carcinoma cells contain a stable and constitutively active β-Catenin-TCF4 complex in the nuclei and that reintroduction of APC removes β-Catenin from TCF4 and abrogates the transcriptional activation of TCF4. These in vitro findings suggest that constitutive transcription of the TCF target genes, which is caused by the loss of APC function, may be a crucial event in the early transformation of the colonic epithelium. In this study, β-Catenin is also expressed in the nuclei in human colorectal adenomas and carcinomas including HGINs, as reported previously [26,27,39]. The elevated nuclear expression of both TCF4 and β-Catenin is in line with their theorized roles in early colorectal carcinogenesis.

Multiple correlations of expressions among p-c-Jun, TCF4 and β-Catenin, and their colocalization within nucleus shown by immunofluorescence analysis are consistent with two recent in vitro studies [17,18]. One shows that p-c-Jun interacts with TCF4 to form a ternary complex containing c-Jun, TCF4 and β-Catenin and that p-c-Jun and TCF4 cooperatively activate the *c-jun *promoter in a β-Catenin-dependent manner [17]. The other shows that the armadillo repeat domain of β-Catenin physically associates with the DNA-binding domain of c-Jun and that β-Catenin target genes, *cyclin D1 *and *c-myc*, are transcriptionally activated by c-Jun through TCF-binding site [18]. Both studies show these interactions cooperatively enhance their transcriptional activities of promoters containing AP-1 and TCF-binding sites including *c-jun *promoter itself, which can promote a positive feedback loop for c-Jun expression, consistent with their correlations observed in human colorectal tumors. Significantly correlated expression of p-c-Jun and TCF4 with MMP7, which has functional AP-1 site and TCF4 site in its promoter [19], may support the hypothesis that interaction of p-c-Jun, TCF4 and β-Catenin enhance transcriptional activity and that the interaction is also important in human colorectal carcinogenesis.

## Conclusion

These results suggest that nuclear expression of p-c-Jun, TCF4 and β-Catenin have important roles in human colorectal tumor development and that p-c-Jun may play a pivotal role in the earlier stages of this tumor development. Small molecule inhibitors of JNKs have been shown to inhibit c-Jun phosphorylation and the phosphorylation-dependent interactions between c-Jun and TCF4 [17]. AP-1 blockade by a c-Jun dominant-negative mutant has been shown to inhibit growth of cancer cells includes colon cancer cells [40,41]. Such inhibitors may have potential for use as preventive and therapeutic strategies against colorectal cancers.

## Competing interests

The authors declare that they have no competing interests.

## Authors' contributions

KT participated in the design of the study, performed the research, and drafted the manuscript. IK conceived of, designed and supervised the study, and critically revised the manuscript. YS participated in collection of clinical data. YO helped immunofluorescence analysis and pathological determination of tumor content and participated in reviewing manuscript. TI and YM participated in the pathological data evaluation. TS provided surgical specimens and contributed to critical discussion. HD-A supervised the study and participated in reviewing the manuscript. All authors read and approved the final manuscript.

## Pre-publication history

The pre-publication history for this paper can be accessed here:



## Supplementary Material

Additional file 1 **Supplemental Table S1**: Nuclear expression of p-c-Jun, TCF4 β-Catenin and MMP7 in tumor compared with adjacent normal colorectal epithelia by using immunohistochemistry (IHC) scores. Click here for file

Additional file 2 **Supplemental Table S2**: Correlation between staining intensity and percentage of positive cells (percentage score) in all colorectal tumors and adjacent normal epithelia (n = 68, respectively). Click here for file

Additional file 3 **Supplemental Table S3: **Nuclear expression of p-c-Jun, TCF4, β-Catenin and MMP7 in tumor compared with adjacent normal colorectal epithelia by using percentage scores. Click here for file

Additional file 4 **Supplemental Figure S1: **Nuclear expression of p-c-Jun, TCF4 and β-Catenin in adenomas (n = 19), HGINs (n = 14) and adenocarcinomas (n = 35) by using percentage of positive cells (percentage score). Only p-c-Jun expression was significantly different. Adenomas and HGINs showed significantly higher percentage scores than adenocarcinomas (p = 0.01). Horizontal lines, median; boxes, 25% to 75% range; brackets, 10% to 90% range; circles, points outside the 10% to 90% range. *p < 0.05 by Kruskal-Wallis test. Click here for file

Additional file 5 **Supplemental Figure S2: **Relationship between nuclear expression of p-c-Jun, TCF4 and β-Catenin and the pT and pN stages in adenocarcinomas (n = 35) by using percentage score. a) p-c-Jun expression was significantly negatively correlated with the pT stage progression (T1-2, n = 11; T3-4, n = 24). b) No significant correlation was observed between p-c-Jun, TCF4 and β-Catenin expression, and the pN stage (N0, n = 20; N1-3, n = 15). Horizontal lines, median; boxes, 25% to 75% range; brackets, 10% to 90% range; circles, points outside the 10% to 90% range. *p < 0.05 by Mann-Whitney U test. Click here for file
