# Supplementary material for: Clinicopathological significance of expression of p-c-Jun, TCF4 and beta-Catenin in colorectal tumors
Source: BMC Cancer. 2008 Nov 8;8:328. doi: 10.1186/1471-2407-8-328 (PMC2585585; doi:10.1186/1471-2407-8-328)
Supplement: Additional file 1 — Supplemental Table S1: Nuclear expression of p-c-Jun, TCF4 β-Catenin and MMP7 in tumor compared with adjacent normal colorectal epithelia by using immunohistochemistry (IHC) scores. [file 1471-2407-8-328-S1.pdf]

Additional file 1-Supplemental Table S1.
Nuclear expression of p-c-Jun, TCF4, β-Catenin and MMP7 in tumor compared with adjacent normal colorectal epithelia

Tumor type	n	p-c-Jun			TCF4			β-Catenin			MMP7		
		Normal /	Tumor	*p*	Normal /	Tumor	*p*	Normal /	Tumor	*p*	Normal /	Tumor	*p*
Adenoma	19	10 (20 ± 28) /	25 (45 ± 50)	0.02*	70 (99 ± 79) /	150 (136 ± 64)	0.048*	30 (29 ± 21) /	70 (75 ± 55)	0.002*	10 (22 ± 33) /	120 (112 ± 66)	0.0004*
HGIN	14	0 (12 ± 21) /	30 (33 ± 24)	0.02*	104 (131 ± 104) /	180 (167 ± 69)	0.2	35 (56 ± 57) /	103 (99 ± 59)	0.004*	25 (27 ± 31) /	95 (111 ± 68)	0.002*
Carcinoma	35	1 (10 ± 31) /	15 (21 ± 26)	0.0006*	150 (128 ± 86) /	143 (133 ± 73)	0.9	30 (33 ± 31) /	100 (116 ± 73)	<0.0001*	0 (20 ± 26) /	30 (51 ± 54)	0.001*

Data are expressed as immunohistochemistry (IHC) scores; median (mean ± SD).

*$p < 0.05$ by Wilcoxon matched pairs signed ranks test.
