# Supplementary material for: Clinicopathological significance of expression of p-c-Jun, TCF4 and beta-Catenin in colorectal tumors
Source: BMC Cancer. 2008 Nov 8;8:328. doi: 10.1186/1471-2407-8-328 (PMC2585585; doi:10.1186/1471-2407-8-328)
Supplement: Additional file 2 — Supplemental Table S2: Correlation between staining intensity and percentage of positive cells (percentage score) in all colorectal tumors and adjacent normal epithelia (n = 68, respectively). [file 1471-2407-8-328-S2.pdf]

Additional file 2–Supplemental Table S2
Correlation between staining intensity and percentage of positive cells (percentage score) in all colorectal tumors and adjacent normal epithelia (n = 68, respectively)

Factor	Tissue	rs	p	
p-c-Jun	Normal	0.95	<0.0001	*
p-c-Jun	Tumor	0.63	<0.0001	*
TCF4	Normal	0.78	<0.0001	*
TCF4	Tumor	0.52	<0.0001	*
β-Catenin	Normal	0.81	<0.0001	*
β-Catenin	Tumor	0.63	<0.0001	*
MMP7	Normal	0.89	<0.0001	*
MMP7	Tumor	0.35	0.01 *	

*p < 0.05 by Spearman's rank correlation test.
