# Supplementary material for: Clinicopathological significance of expression of p-c-Jun, TCF4 and beta-Catenin in colorectal tumors
Source: BMC Cancer. 2008 Nov 8;8:328. doi: 10.1186/1471-2407-8-328 (PMC2585585; doi:10.1186/1471-2407-8-328)
Supplement: Additional file 3 — Supplemental Table S3: Nuclear expression of p-c-Jun, TCF4, β-Catenin and MMP7 in tumor compared with adjacent normal colorectal epithelia by using percentage scores. [file 1471-2407-8-328-S3.pdf]

Additional file 3 –Supplemental Table S3

Nuclear expression of p–c–Jun, TCF4, β–Catenin and MMP7 in tumor compared with adjacent normal colorectal epithelia by using percentage score

Tumor type	n	p–c–Jun		TCF4		β–Catenin		MMP7	
		Normal / Tumor	*p*	Normal / Tumor	*p*	Normal / Tumor	*p*	Normal / Tumor	*p*
Adenoma	19	10 (17 ± 19) / 20 (26 ± 21)	0.04*	60 (54 ± 32) / 75 (69 ± 25)	0.04 *	30 (27 ± 18) / 60 (54 ± 27)	0.003*	10 (16 ± 21) / 50 (47 ± 19)	0.0006*
HGIN	14	0 (12 ± 21) / 25 (25 ± 14)	0.02*	73 (61 ± 34) / 90 (80 ± 20)	0.04 *	35 (43 ± 32) / 69 (61 ± 24)	0.01 *	20 (20 ± 27) / 55 (53 ± 24)	0.002 *
Carcinoma	35	3 (8 ± 16) / 10 (14 ± 16)	0.01*	85 (66 ± 36) / 90 (76 ± 29)	0.06	30 (30 ± 25) / 75 (67 ± 26)	<0.0001*	0 (19 ± 26) / 20 (25 ± 23)	0.08

Data are expressed as percentage score; median (mean ± SD).

*p < 0.05 by Wilcoxon matched pairs signed ranks test.
