# Supplementary figures and images for: Clinicopathological significance of expression of p-c-Jun, TCF4 and beta-Catenin in colorectal tumors
Source: BMC Cancer. 2008 Nov 8;8:328. doi: 10.1186/1471-2407-8-328 (PMC2585585; doi:10.1186/1471-2407-8-328)

p-c-Jun

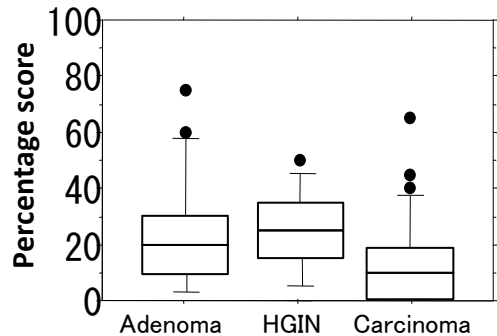 $p = 0.01^*$ 

TCF4

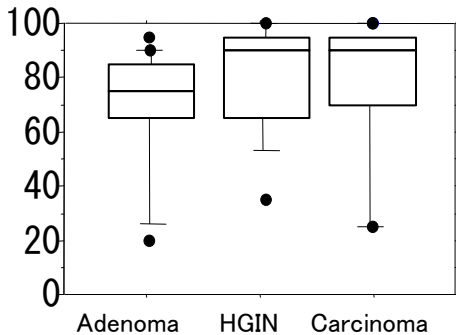 $p = 0.1$  $\beta$ -Catenin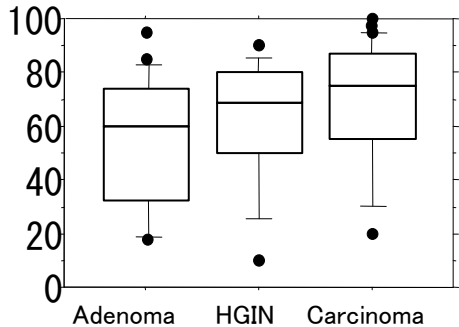 $p = 0.2$

Supplement: Additional file 4 — Supplemental Figure S1: Nuclear expression of p-c-Jun, TCF4 and β-Catenin in adenomas (n = 19), HGINs (n = 14) and adenocarcinomas (n = 35) by using percentage of positive cells (percentage score). Only p-c-Jun expression was significantly different. Adenomas and HGINs showed significantly higher percentage scores than adenocarcinomas (p = 0.01). Horizontal lines, median; boxes, 25% to 75% range; brackets, 10% to 90% range; circles, points outside the 10% to 90% range. *p < 0.05 by Kruskal-Wallis test. [file 1471-2407-8-328-S4.pdf]

(a)

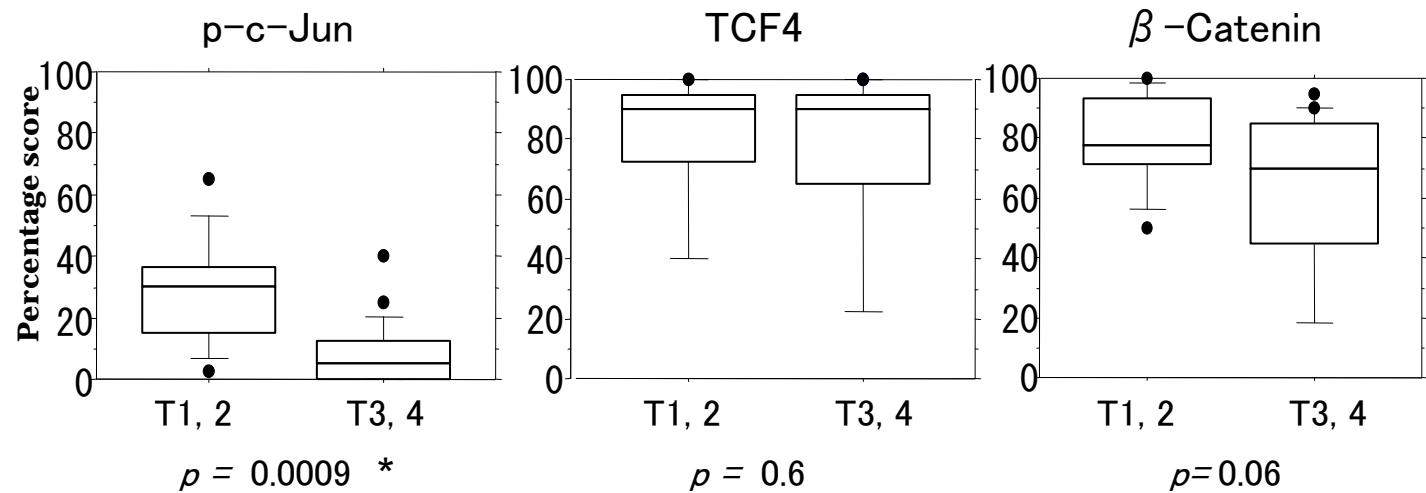

(b)

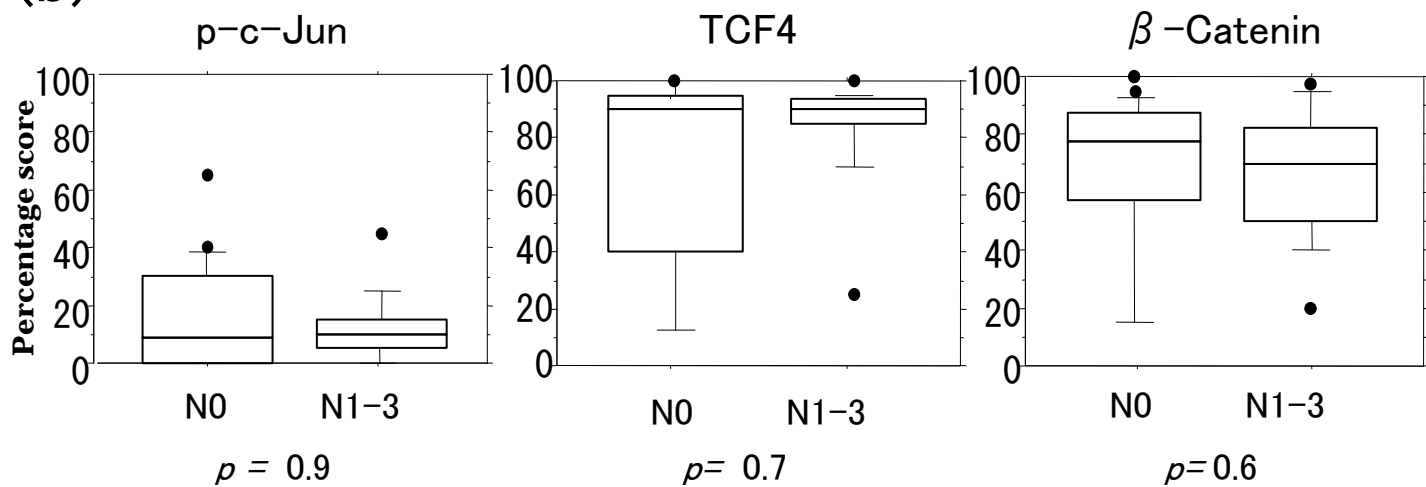

Supplement: Additional file 5 — Supplemental Figure S2: Relationship between nuclear expression of p-c-Jun, TCF4 and β-Catenin and the pT and pN stages in adenocarcinomas (n = 35) by using percentage score. a) p-c-Jun expression was significantly negatively correlated with the pT stage progression (T1-2, n = 11; T3-4, n = 24). b) No significant correlation was observed between p-c-Jun, TCF4 and β-Catenin expression, and the pN stage (N0, n = 20; N1-3, n = 15). Horizontal lines, median; boxes, 25% to 75% range; brackets, 10% to 90% range; circles, points outside the 10% to 90% range. *p < 0.05 by Mann-Whitney U test. [file 1471-2407-8-328-S5.pdf]
